# Silica inhalation altered telomere length and gene expression of telomere regulatory proteins in lung tissue of rats

**DOI:** 10.1038/s41598-017-17645-y

**Published:** 2017-12-11

**Authors:** Mohammad Shoeb, Pius Joseph, Vamsi Kodali, Gul Mustafa, Breanne Y. Farris, Christina Umbright, Jenny R. Roberts, Aaron Erdely, James M. Antonini

**Affiliations:** Health Effects Laboratory Division, National Institute for Occupational Safety and Health, Morgantown, WV USA

## Abstract

Exposure to silica can cause lung fibrosis and cancer. Identification of molecular targets is important for the intervention and/or prevention of silica-induced lung diseases. Telomeres consist of tandem repeats of DNA sequences at the end of chromosomes, preventing chromosomal fusion and degradation. Regulator of telomere length-1 (RTEL1) and telomerase reverse transcriptase (TERT), genes involved in telomere regulation and function, play important roles in maintaining telomere integrity and length. The goal of this study was to assess the effect of silica inhalation on telomere length and the regulation of RTEL1 and TERT. Lung tissues and blood samples were collected from rats at 4, 32, and 44 wk after exposure to 15 mg/m^3^ of silica × 6 h/d × 5 d. Controls were exposed to air. At all-time points, RTEL1 expression was significantly decreased in lung tissue of the silica-exposed animals compared to controls. Also, significant increases in telomere length and TERT were observed in the silica group at 4 and 32 wk. Telomere length, RTEL1 and TERT expression may serve as potential biomarkers related to silica exposure and may offer insight into the molecular mechanism of silica-induced lung disease and tumorigeneses.

## Introduction

It is estimated that approximately 1.7 million U.S. workers are exposed to respirable crystalline silica in a number of occupations and industries, including mining, construction, and sandblasting^1^. Silicosis is an irreversible chronic lung disease characterized by inflammation, alveolar proteinosis, and diffuse fibrosis, resulting in progressive restrictive lung function after prolonged exposure to crystalline silica^2^. Other disorders, such as lung cancer, tuberculosis, chronic renal disease, and autoimmune diseases, also have been observed after occupational exposure to crystalline silica^3,4^. Silica exposure in animal toxicology studies resulted in an initial acute inflammatory response that was followed by the progression and eventual development of pulmonary fibrosis and alveolar proteinosis, confirming findings in humans^5–7^. Silica has been shown to be cytotoxic to phagocytes^8–10^ and not readily cleared from the airspaces after pulmonary deposition, causing its relatively long persistence in lung structures. Because of this, the continual recruitment of phagocytes (e.g., macrophages and neutrophils) into lung airspaces and the resulting ongoing production and secretion of inflammatory cytokines, reactive oxygen species (ROS), and hydrolytic enzymes have been shown to damage lung tissues and cells and produce the chronic lung injury and inflammation associated with silica exposure^5–7,11^.

Because silicosis is an irreversible and life-threatening lung disease, the National Institute for Occupational Safety and Health has recommended that sensitive and practical methodologies be developed to predict disease outcome before the appearance of clinical symptoms of silicosis^12^. The measurement of telomere length and/or the expression of proteins that regulate telomeres may serve as potential biomarkers to assess past silica exposure and possibly predict future development of silicosis. Telomeres are nucleoprotein structures composed of simple repetitive structures of DNA sequences (TTAGGG)_n_ that stabilize ends of chromosomes by preventing DNA degradation and preserving genetic information^13^. Environmental and cellular factors impact telomeres throughout one’s lifespan^14^. Telomeres generally shorten with age, and their length may be influenced by stressors, such as diet, physical activity, chronic inflammation, and occupational and environmental exposures^15–17^. Importantly, changes in telomere length have been associated with an increased risk of lung cancer development^18,19^.

Telomerase activity is primarily responsible for maintaining telomere integrity by adding TTAGGG repeats on chromosomal ends to compensate for the continual reduction in telomere length due to aging^20^. A correlation between telomerase activity and mRNA expression of telomerase reverse transcriptase (TERT), a catalytic subunit of telomerase, has been observed^21^. Thus, TERT has been commonly used as a measure of telomerase activity. Importantly, increased mutations affecting telomere length and function have been observed in patients with idiopathic pulmonary fibrosis, a chronic lung disease^22,23^. In addition, regulator of telomere length-1 (RTEL1), a protein involved in telomere length regulation and protection of chromosomes from damage, also was measured^24^. RTEL1 has been observed to play an important role in regulating telomere length, maintenance, and repair^25–27^.

The goal of the current study was to assess the effect of silica inhalation exposure on telomere length and the regulation of RTEL1 and TERT gene expression involved in telomere maintenance in the blood and lung tissue *in vivo*. Banked tissue samples from previous studies were collected from male Fischer 344 rats that had been exposed by inhalation to 15 mg/m^3^ of silica for 6 h/d for 5 d^11,28,29^. Rats were sacrificed at 4, 32, and 44 wk after the 5-d exposure. Total RNA was isolated, and expression of RTEL1 and TERT regulatory genes was assessed. Genomic DNA (gDNA) was isolated to measure telomere length in exposed lung tissue. It is our hypothesis that changes in telomere length and the genes involved in telomere regulation may serve as potential biomarkers related to silica exposure and also may offer insight into the molecular mechanism of silica-induced lung diseases, such as fibrosis and tumorigenesis.

## Results and Discussion

Inhalation of silica is a known occupational hazard characterized by the progression from pulmonary inflammation and cellular injury to fibrosis, and the possible development of lung cancer^1,4^. Previously, it was reported that silica inhalation (15 mg/m^3^ × 6 h/d × 5 d) resulted in lung injury (elevated LDH response) and inflammation (increased number of BALF PMNs) in rats that progressed up to 44 wk after exposure as determined by histopathological analysis and assessment of recovered BALF from exposed rats (Table 1;^11,28,29^). Silica inhalation increased molecular markers of inflammation (e.g., MCP-1) at all time points post-exposure (Table 1). In addition, increased type II pneumocyte hyperplasia and lung fibrosis were the major histological changes observed at 32 and 44 wk post-silica exposure. However, lung fibrosis was more severe at 44 wk compared to 32 wk, and no histological changes was noticed at 4 wk post-exposure (Table 1). In the current investigation, banked lung tissue and blood samples collected from rats exposed to silica by inhalation from these previous studies^11,28,29^ were analyzed for effects on telomere length and the regulation of different genes involved in telomere maintenance.

**Table 1:** Lung Injury and Inflammation after Silica Inhalation Exposure.

Parameter	4 weeks^a^		32 Weeks^b^		44 Weeks^c^	
	Air	Silica	Air	Silica	Air	Silica
BALF LDH (U/L BALF)	70.75 ± 7.03	111.38 ± 13.02*	86.14 ± 8.86	280.75 ± 10.06*	64.14 ± 9.51	157.75 ± 12.41*
BALF PMN (10^6^)	0.83 ± 0.06	1.41 ± 0.11*	1.61 ± 0.69	12.45 ± 0.93*	0.57 + 0.03	2.27 ± 0.27*
BALF MCP-1 (pg/ml BALF)	28.55 ± 12.70	39.15 ± 14.41	28.18 ± 3.05	460.23 ± 59.23*	31.99 ± 2.13	175.29 ± 31.57*
Lung Fibrosis- Trichrome Stain	−	−	−	+	−	+

Note. Data was modified from previously published studies: ^a^Sellamuthu *et al*.^11^; ^b^Sellamuthu *et al*.^28^; ^c^Sellamuthu *et al*.^29^; *significantly different from corresponding air control (p < 0.05); Trichrome: No fibrosis (−), Positive for fibrosis (+).

Oxidative stress and the resultant production of ROS have been observed to be a primary mechanism by which silica exposure damages lung tissue and cells^5,7,9^. Importantly, oxidative stress also has been proposed as an important mechanism in causing potential alterations in telomere length^30^. Telomeres are complexes of tandem repetitive structures of DNA (5′-TTAGGG-3′) on the ends of chromosomes that prevent DNA degradation and preserve genetic information^13,20^. Telomeres shorten with age, and their length can be influenced by a lifetime of exposure to inhaled environmental and occupational substances^15,16^. Variable responses in telomere length changes have been observed after exposure to different inhaled pollutants^31^.

**Figure 1:**
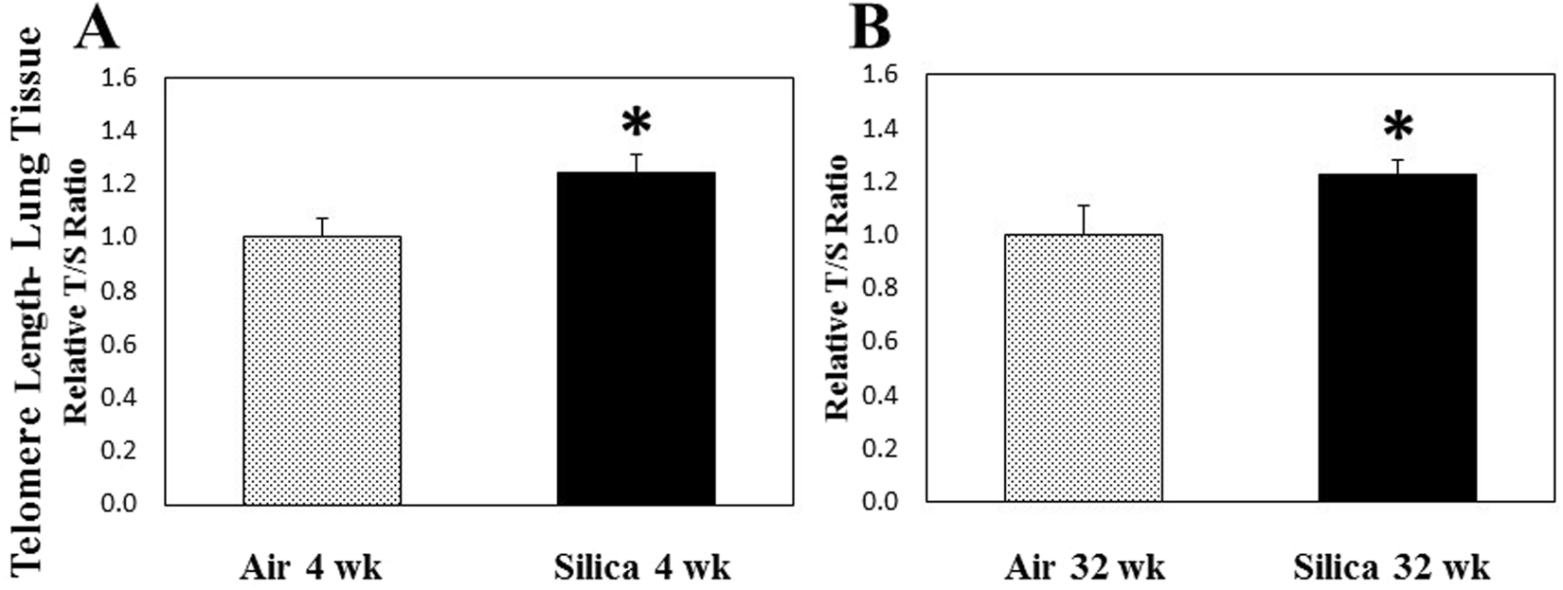
Telomere length ratio of lung tissue gDNA after a 1-wk silica inhalation exposure (15 mg/m^3^ for 6 h/d for 5 d). Lung tissues were collected at (**A**) 4 and (**B**) 32 wk after the 5-d exposure. Rats exposed to filtered air served as the controls. (n = 8; values are means ± standard error; *significantly different from corresponding air control, p < 0.05).

**Figure 2:**
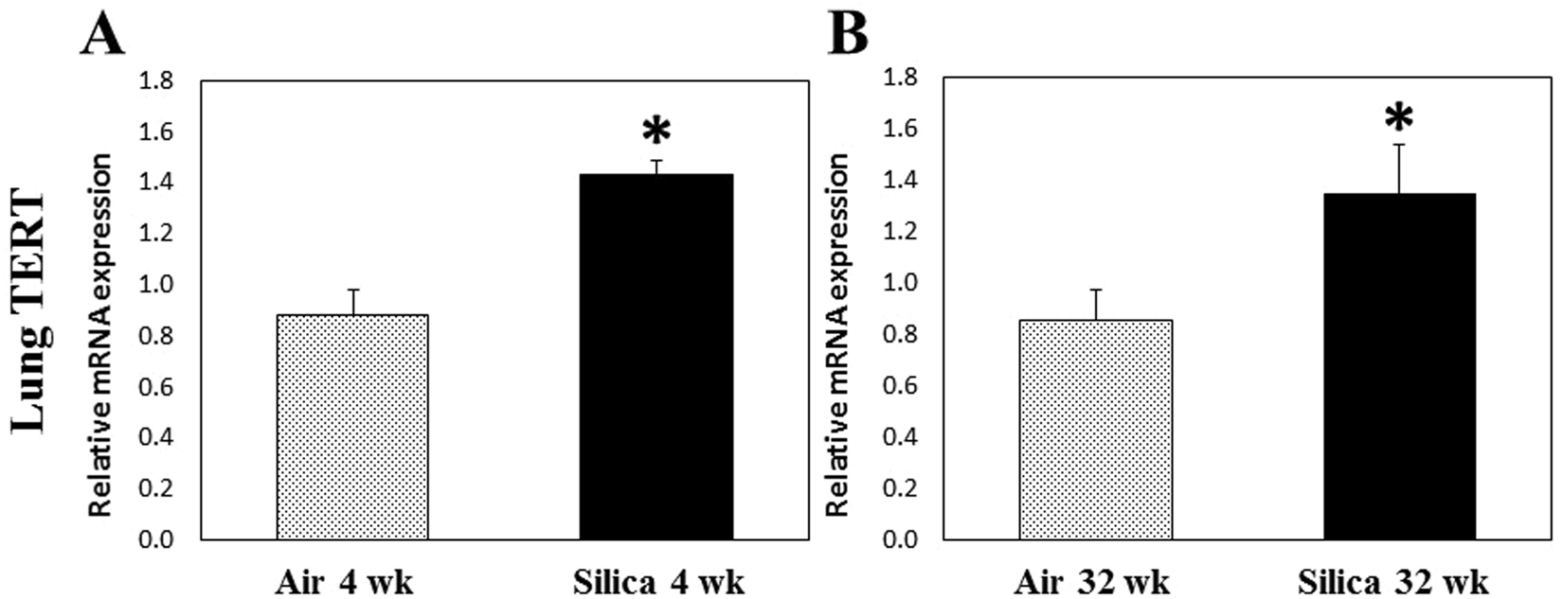
TERT expression in lung tissue cDNA after a 1-wk silica inhalation exposure (15 mg/m^3^ for 6 h/d for 5 d). Lung tissues were collected at (**A**) 4 and (**B**) 32 wk after the 5-d exposure. Rats exposed to filtered air served as the controls. (n = 8; values are means ± standard error; *significantly different from corresponding air control, p < 0.05).

Both telomere length ratio (Fig. 1) and TERT gene expression (Fig. 2) were significantly elevated in lung tissue at 4 and 32 wk after a 5-d silica exposure compared to air controls. Telomere integrity is maintained by telomerase activity by which TTAGGG repeats are added on the ends of chromosomes to offset continual degradation of telomeres that occurs with aging^20^. Elevated telomerase activity could result in increased telomere length and is dependent on the expression of TERT, through which telomeric repeats are synthesized by reverse-transcribing the RNA template. Mutations in TERT have been observed in chronic lung diseases, such as idiopathic pulmonary fibrosis^22,23^. Kim *et al.* ^32^ demonstrated that lung tissue from rats treated with silica (40 mg/kg) by intratracheal instillation had a significant increase in telomerase activity at 24 h, 1 wk, and 8 wk after treatment. In their study, it was concluded that the early increase in telomerase activity was due to the generation of ROS and inflammation caused by silica treatment. Another possible explanation for the increase in telomere length in the lung tissue at 4 and 32 wk may be due to the presence of more cells in the lungs caused by the influx of a significantly greater number of inflammatory cells that was observed after silica inhalation in the previous studies^11,28,29^. Furthermore, increases in telomere length were observed in biological samples collected after acute exposures to metal-rich particulates in steel workers^33^ and elemental carbon associated with particulate matter inhalation^31^. In addition, Colicino *et al*.^34^ observed an increase in telomere length of peripheral leukocytes after a year-long exposure to traffic-related air pollution. These studies also implicated oxidative stress and inflammation as potential causes related to the observed alterations in telomere length. Using an *in vitro* system, Borghini *et al*.^35^ observed oxidative DNA damage in human lung epithelial cells and telomere length shortening after exposure to multi-walled carbon nanotubes, possibly implicating particle exposure in the development of DNA instability.

**Figure 3:**
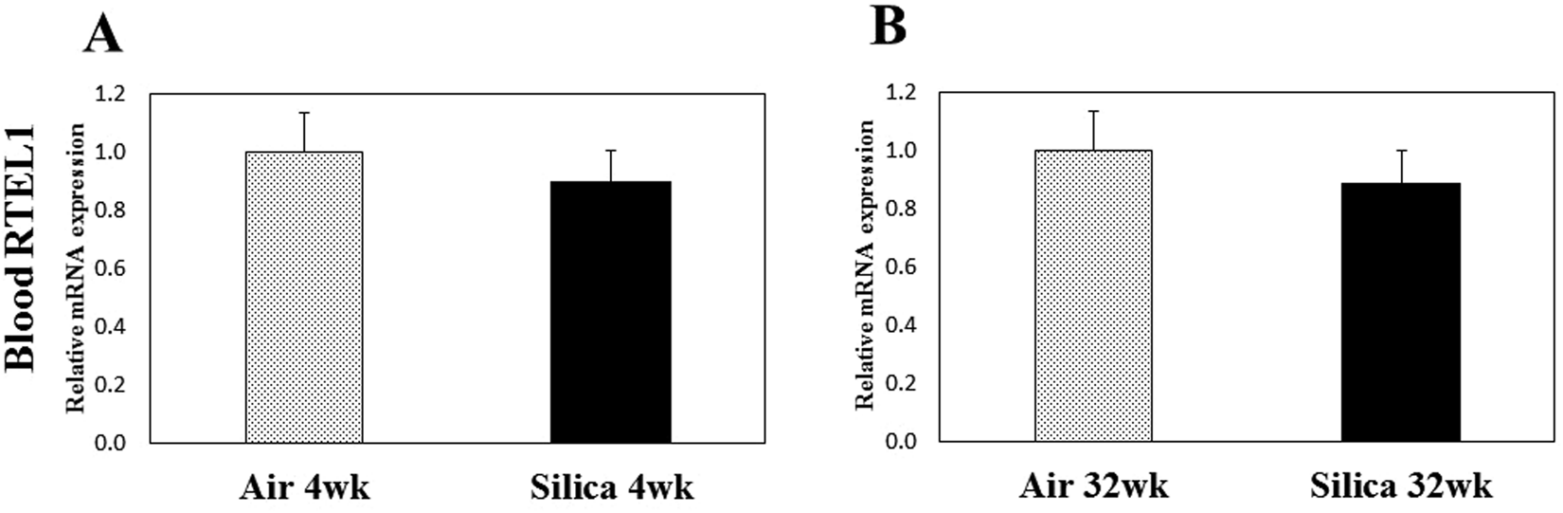
RTEL1 expression in blood cDNA after a 1-wk silica inhalation exposure (15 mg/m^3^ for 6 h/d for 5 d). Blood was collected (**A**) 4 and (**B**) 32 wk after the 5-d exposure. Rats exposed to filtered air served as the controls; n = 8; values are means ± standard error).

**Figure 4:**
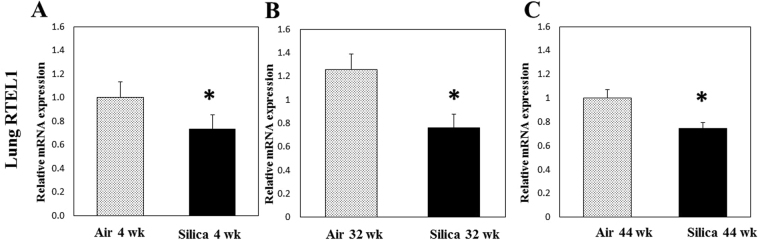
RTEL1 expression in lung tissue cDNA after a 1-wk silica inhalation exposure (15 mg/m^3^ for 6 h/d for 5 d). Lung tissues were collected at (**A**) 4, (**B**) 32, and (**C**) 44 wk after the 5-d exposure. Rats exposed to filtered air served as the controls. (n = 8; values are means ± standard error; *significantly different from corresponding air control, p < 0.05).

As a measure of telomere length stability and regulation, gene expression of RTEL1 was determined in blood (distal site of exposure) and lung tissue (site of exposure and toxicity) of exposed animals in the current study. RTEL1, a DNA helicase, is imperative in the maintenance of the telomere-loop (T-Loop) structure of telomeres^24^. Mutations or inhibition of RTEL1 have been associated with changes in telomere length^25–27^. RTEL1 has been proposed to disassemble T-Loop structures and counteract telomere G4-DNA structures to allow efficient telomere replication and promote telomere stability^26^. Deletion and inactivation of RTEL1 have resulted in telomere fragility and loss as well as changes in telomere heterogeneity. Loss of RTEL1 has been observed to cause rapid alterations in mean telomere length, with corresponding increases in both short and long telomeres^26^. In blood, cDNA recovered at 4 and 32 wk after a 5-d exposure had no change in RTEL1 expression compared to air controls (Fig. 3). However, RTEL1 gene expression was significantly decreased in lung tissue compared to air controls at 4, 32, and 44 wk after a 5-d exposure to silica (Fig. 4). Another reasonable explanation for the increases in telomere length in lung tissue observed in the current study would be that silica inhalation caused a significant reduction in RTEL1 expression which resulted in telomere instability, thus altering telomere length. The resulting chromosomal instability may disrupt genomic integrity that could result in cancer initiation and tumor development. Both increases^19,36^ and decreases^18,37^ in telomere length have been associated with increased risk of various cancer types, including lung cancer.

In conclusion, telomere length changes were observed at the site of particle deposition and toxicity (lungs), but not at an extra-pulmonary site (blood) after inhalation exposure to silica. In addition, gene expression of specific proteins involved in telomere regulation were altered in lung tissue after silica exposure. The changes in length and regulation of telomeres caused by silica inhalation may result in chromosomal instability, possibly resulting in adverse chronic health effects. Previously, we have observed that stainless steel welding fume composed of carcinogenic metals (e.g., chromium and nickel) significantly increased telomere length in peripheral blood mononuclear cells^17^. Furthermore, pulmonary aspiration^38^ and inhalation^39^ of stainless steel welding fume promoted lung tumorigenesis in an animal model that was consistent with the findings in a review of cancer epidemiology in the scientific literature, concluding welders may be at an increased risk of lung cancer^40^. Based on the findings by our group and others^31,33,34^, the inhalation effects of particle exposure on telomere length and regulatory genes and the potential associated long-term toxicological outcomes (e.g., tumorigenesis, carcinogenesis) need further study. The measurement of telomere length in biological samples (e.g., lung biopsy, lung cells and BALF, serum, peripheral blood mononuclear cells) may serve as a potential biomarker in the assessment of toxicity associated with inhaled particle exposure.

## Materials and Methods

### Animals

Analysis of tissue samples for the current study was performed on banked lung and blood samples collected from rats exposed in a previous silica exposure study that has resulted in multiple publications^11,28,29^. In the study, male Fischer 344 rats (pathogen-free CDF strain; 2–3 months old; Charles River Laboratories; Wilmington, MA, USA) were used. Upon arrival, the rats were acclimated for approximately 10 days and were provided HEPA-filtered air, irradiated Teklad 2918 diet, and tap water *ad libitum*. Two rats were housed per cage under controlled room temperature (22–25 °C) and humidity (40–60%). All animal procedures used during the study were reviewed and approved by the National Institute for Occupational Safety and Health’s Animal Care and Use Committee. The animal facilities are specific pathogen-free, environmentally-controlled, and accredited by the AAALAC International (Frederick, MD, USA). All methods were performed in accordance with the relevant guidelines and regulations by the National Institute for Occupational Safety and Health.

### Inhalation Exposure of Rats to Silica Aerosol

An aerosol containing crystalline silica particles of respirable size (mass median aerodynamic diameter = 1.6 μm; geometric standard deviation = 1.6) was generated from a bulk supply of Min-U-Sil 5 silica (U.S. Silica, Berkeley Springs; WV, USA). Control animals were exposed to filtered air. Rats were euthanized at 4, 32, and 44 wk after the 5-d exposure. Tissue samples were analyzed from rats exposed to 15 mg/m^3^ of silica for 6 h/d for 5 d.

Rats in all groups were euthanized following an intraperitoneal injection of 100–300 mg sodium pentobarbital/kg body weight (Fort Dodge Animal Health; Fort Dodge, IA, USA). Blood was collected directly from the abdominal aorta from all rats and transferred to Vacutainer blood tubes containing EDTA as an anticoagulant (Becton-Dickinson; Franklin Lakes, NJ, USA). Bronchoalveolar lavage fluid (BALF) was collected from right lungs as previously described^11,28,29^ to assess lung injury (lactate dehydrogenase- LDH activity), cellular inflammation (polymorphonuclear neutrophil number- PMNs), and pro-inflammatory cytokine expression (e.g., monocyte chemoattractant protein-1- MCP-1). Non-lavaged right lungs were collected from all rats and assessed for lung fibrosis (positive trichrome staining) or processed as described in the following sections.

### RNA isolation from Blood and Lung Tissue Samples

Based on sample availability from previous studies, cDNA from lung tissue was isolated at 4, 32, and 44 wk after the 5-d silica exposure; RNA was isolated and reverse transcribed to synthesize cDNA from lung and blood at 4 and 32 wk after the 5-d silica exposure.

Total RNA was isolated from the right lung of the control and silica-exposed rats using an RNeasy Fibrous Tissue Mini Kit (Qiagen Inc.; Valencia, CA, USA) according to kit instructions. Briefly, 25–30 mg of lung tissue was homogenized in buffer RLT and two 2.4 mm Zirconia beads (BioSpec Products Inc.; Bartlesville, OK, USA) using a mini beadbeater-8 (BioSpec Products Inc.) for 20 sec. The tissue homogenate was centrifuged at 10,000 × g for 10 min at room temperature, and the RNA present in the supernatant was extracted and purified using RNeasy columns.

Total RNA from the rat blood samples was isolated using the Mouse Ribopure Blood RNA Isolation Kit (Ambion, Inc.; Austin, TX, USA). The RNA isolated was digested with RNase-free DNase and further purified using the RNeasy Mini Kit (Qiagen Inc.). Globin mRNA was present in abundance in the blood; therefore depletion of globin mRNA from the blood RNA samples was performed using the Globinclear-Mouse/Rat Globin mRNA Removal Kit (Ambion, Inc.). RNA was quantitated by UV spectrophotometry. RNA was reverse transcribed using random hexamers (Applied Biosystems, Foster City, CA, USA) and Superscript III (Invitrogen; Carlsbad, CA, USA). cDNA was used for gene expression. Hypoxanthine-guanine phosphoribosyltranferase (HPRT) was used as the endogenous control. Some portions of the right lung tissue was immediately kept in RNA later (Ambion, Inc.) and stored at −80 °C for other studies.

### RTEL1 and TERT Analysis

RTEL1 and TERT mRNA levels were determined by quantitative PCR (qPCR). Gene expression was determined by standard 96-well technology using the StepOne Plus (Applied Biosystems; Foster City, CA, USA) with pre-designed Assays-on-Demand TaqMan probes and primers including RTEL1 (Rn01220420_m1) and TERT (Rn01409457_m1) (Thermo Fisher Scientific, Waltham, MA, USA). Using 96 well plates, cDNA was used for gene expression. HPRT was used as the endogenous control.

### gDNA Isolation and Telomere Length Analysis by qPCR

gDNA was extracted from the lung tissue (stored in −80 °C in RNA later) at 4 and 32 wk after the 5-d silica exposure using DNeasy Blood & Tissue Kit (Qiagen Sciences Inc.; Germantown, MD, USA). DNA concentration was measured using the Nano-Drop 2000 spectrophotometer. Samples were diluted to a final concentration of 30 ng/1.2 μl to measure telomere length. Quantitative PCR was performed using the SYBR Select Master Mix (Life Technologies; Carlsbad, CA, USA) with a step one plus real time PCR system (Applied Biosystems, Foster City, CA, USA). The parameters used were as follows: 95 °C for 10 min (enzyme activation), 95 °C for 15 sec (denaturing), and 60 °C for 60 sec (annealing), 60 cycles. Primers used were as follows: Tel rat-F 5′-GGT TTT TGA GGG TGA GGG TGA GGG TGA GGG TGA GGG t-3′, and Tel rat-R 5′-TCC CGA CTA TCC CTA TCC CTA TCC CTA TCC CTA TCC CTA- 3′; AT1 rat-F 5′-ACG TGT TCT CAG CAT CGA CCG CTA CC-3′ and AT1 rat-R 5′-AGA ATG ATA AGG AAA GGG AAC AAG AAG CCC-3′ (Invitrogen Corporation). The relative telomere length was measured by comparing the ratio of telomere repeat copy number (T as Tel1) and single gene copy number (S as AT1), expressed as telomere length (T/S) ratio. Each individual values obtained by qPCR were processed through the formula T/S = 2¬^−^ΔCT, where ΔCT = CTtelomere – CTAT1. This ratio was then compared with the ratio of the reference DNA. Each DNA sample collected was measured in duplicate.

### Statistical analysis

Statistical differences between the silica and air groups within a time point were compared using a one-way ANOVA. Post hoc comparison were made with the Fisher’s Least Significant Difference Test. Values represent means ± standard errors. Criterion of significance was set at p < 0.05.

### Disclaimer

The findings and conclusions in this report are those of the authors and do not necessarily represent the views of the National Institute for Occupational Safety and Health.
